# De Novo Design of Large Polypeptides Using a Lightweight Diffusion Model Integrating LSTM and Attention Mechanism Under Per-Residue Secondary Structure Constraints

**DOI:** 10.3390/molecules30051116

**Published:** 2025-02-28

**Authors:** Sisheng Liao, Gang Xu, Li Jin, Jianpeng Ma

**Affiliations:** 1 School of Life Sciences, Fudan University, Shanghai 200433, China; ssliao22@m.fudan.edu.cn (S.L.); lijin@fudan.edu.cn (L.J.); 2 Multiscale Research Institute of Complex Systems, Fudan University, Shanghai 200433, China; xugang@fudan.edu.cn; 3 Zhangjiang Fudan International Innovation Center, Fudan University, Shanghai 200433, China; 4 Shanghai AI Laboratory, Shanghai 200233, China; 5 State Key Laboratory of Genetic Engineering, Human Phenome Institute, Center for Evolutionary Biology, and Collaborative Innovation Center for Genetics and Development, Fudan University, Shanghai 200433, China; 6 Research Unit of Dissecting Population Genetics and Developing New Technologies for Treatment and Prevention of Skin Phenotypes and Dermatological Diseases (2019RU058), Chinese Academy of Medical Sciences, Beijing 100730, China

**Keywords:** DDPM, protein design, deep learning, neural network, text generation models, long short-term memory networks (LSTM), attention mechanism

## Abstract

This study presents PolypeptideDesigner (PPD), a novel conditional diffusion-based model for de novo polypeptide sequence design and generation based on per-residue secondary structure conditions. By integrating a lightweight LSTM-attention neural network as the denoiser within a diffusion framework, PPD offers an innovative and efficient approach to polypeptide generation. Evaluations demonstrate that the PPD model can generate diverse and novel polypeptide sequences across various testing conditions, achieving high pLDDT scores when folded by ESMFold. In comparison to the ProteinDiffusionGenerator B (PDG-B) model, a relevant benchmark in the field, PPD exhibits the ability to produce longer and more diverse polypeptide sequences. This improvement is attributed to PPD’s optimized architecture and expanded training dataset, which enhance its understanding of protein structural pattern. The PPD model shows significant potential for optimizing functional polypeptides with known structures, paving the way for advancements in biomaterial design. Future work will focus on further refining the model and exploring its broader applications in polypeptide engineering.

## 1. Introduction

The denoising diffusion probabilistic model (DDPM) [1] and its derivative diffusion-based models [2,3,4,5,6,7] have emerged as a powerful class of generative models, demonstrating superior performance in generating high-quality images, audio, and video. These models have consistently outperformed Generative Adversarial Networks (GANs) and other generative approaches across multiple benchmarks [8,9,10,11,12,13,14,15,16,17]. The superiority of diffusion models can be attributed to generation quality and training stability [1,6,18,19,20,21,22,23,24]. In addition, diffusion models exhibit remarkable flexibility, enabling the development of numerous variants and extensions tailored for specific tasks [17]. A notable example is Imagen, a cutting-edge diffusion-based model developed by Google, which has been specifically adapted for generating images from textual descriptions [4]. Imagen possesses exceptional comprehension capabilities for textual conditions, generating images that exhibit a high degree of concordance with textual descriptions. Another significant advancement is Stable Diffusion, which incorporates Contrastive Language-Image Pre-training (CLIP) techniques [25,26] and operates in a compressed latent space [5], achieving excellent generation quality and stability even with limited computational resources [27,28]. The outstanding performance of diffusion models has aroused widespread interest among researchers, prompting an exploration of their applications across various fields. Particularly noteworthy are the remarkable achievements attained in the field of biology [17,29,30,31,32,33,34,35,36].

Protein structure prediction, a central challenge in structural biology, has witnessed remarkable progress through the integration of deep learning techniques. The field has seen the emergence of several influential models that have advanced prediction capabilities. In 2021, the DeepMind team introduced the AlphaFold 2 (AF2), which incorporates a novel graph-based triangle self-attention mechanism, a gated self-attention mechanism, and an iterative refinement module. This model is capable of processing multimodal data that includes multiple sequence alignment (MSA) [37,38,39,40], residue pairs, and template structure information, significantly enhancing the accuracy of predicting both the backbone and side chain folding states of proteins. Subsequently, based on AF2, the DeepMind team proposed AlphaFold-Multimer (AFM), a model for the structure prediction of multimeric complexes. By integrating cross-chain homology information and modifying the original model architecture and loss functions, AFM achieves a high-accuracy prediction of protein complex structures [41]. Concurrently, independent of AlphaFold, David Baker and his team have successively proposed RoseTTAFold (RF), RoseTTAFold2 (RF2), and RoseTTAFold All-Atom (RFAA) for the prediction of protein and biomolecular complex structures [42,43,44]. These series of models share similar neural network architectural characteristics, all employing a three-track neural network architecture to process multimodal information. In terms of the accuracy of protein structure prediction for both monomers and multimers, RF2 rivals that of AF2 and AFM, respectively. Another notable advancement comes from Meta AI’s ESMFold, which represents a distinct approach in the field. It employs a protein language model with 150 billion parameters to predict protein structures directly from single sequence, bypassing the need for MSA and template structures. This simplification speeds up the inference process, making ESMFold particularly beneficial for large-scale protein structure prediction tasks, especially in metagenomics [45].

Diffusion models have further expanded the frontiers of protein structure prediction, achieving tremendous success in predicting the structures and interactions of cross-biomolecular complexes. A landmark development in this field was AlphaFold 3 (AF3), introduced by DeepMind in 2024, which integrates a diffusion-based model framework to achieve unprecedented precision in predicting biomolecular interactions and structures [30]. The architecture of AF3 mainly comprises three core components, each fulfilling distinct functions: embedding and handling of conditions, denoising and generation, and confidence assessment of predicted structures. Compared to its predecessor AF2, AF3 incorporates several key innovations. The implementation of a diffusion-based framework and the novel Pairformer module has significantly enhanced prediction accuracy. The model streamlines the input processing by eliminating the requirement for explicit MSA representation, thereby reducing computational complexity. Furthermore, AF3 features an expanded and modified input encoding scheme that accommodates a wider range of biomolecular types. Cross-distillation technique for data augmentation has further improved the model’s accuracy and robustness. These advancements position AF3 as a transformative tool in structural biology, demonstrating the immense potential of diffusion models in biomolecular research. AF3 provides novel insights into understanding complex biomolecular interactions, marking a significant leap forward in computational structural biology.

In parallel with their success in protein structure prediction, diffusion models have also made significant strides in protein design. In 2023, Baker et al. proposed a diffusion-based model named RoseTTAFold diffusion (RFdiffusion), which can de novo design protein backbone structures conditioned on ligand spatial geometry [46]. They fine-tuned the neural network of RF, which served as the denoiser for RFdiffusion. To complete the design process, ProteinMPNN [47] is deployed downstream of RFdiffusion to generate the amino acid sequence corresponding to the backbone structure. RFdiffusion has demonstrated exceptional capability in designing protein backbones that precisely accommodate target molecular geometries. Later, Liu et al. independently developed SCUBA-diffusion (SCUBA-D), a co-diffusion model for protein backbone design [48]. This approach differs fundamentally from RFdiffusion as it was trained from scratch using a custom loss function that combines data recovery loss with adversarial loss components. Remarkably, SCUBA-D achieves comparable performance to RFdiffusion in generating novel, experimentally verifiable protein structures. The success of these models demonstrates the strong potential for the extensive application of diffusion models.

Due to the striking similarities between protein sequences and language, it has become increasingly common to treat protein as a special form of text [49,50,51,52,53]. Although diffusion-based models were originally developed for image generation tasks, they have demonstrated remarkable performance in text generation through strategic architectural modifications. A diffusion-based text generation model for designing polypeptide sequences, named ProteinDiffusionGenerator-B (for brevity, it will subsequently be abbreviated as PDG-B in the following text), has been reported in the journal *Chem* [54]. The PDG-B model treats the primary structure sequences and their corresponding per-residue secondary structure sequences as specialized textual images, where each residue is encoded as a discrete pixel. It employs the Imagen framework for diffusion cascade, complemented by an attention-based [55] 1d convolutional U-Net architecture [56,57,58] functioning as the denoiser. This integrated approach demonstrates good performance in generating short polypeptide amino acid sequences.

Building on the diffusion workflow, we introduce PolypeptideDesigner (PPD), an innovative model for conditional polypeptide sequence design and generation. PPD leverages a per-residue secondary structure as the condition needed to steer the generation process, enhancing the diversity and novelty of the generated sequences while maintaining low GPU memory requirements. Our proposed model is designed to achieve multiple objectives: reducing hardware requirements and computational overhead while simultaneously enabling the generation of longer polypeptide chains with enhanced sequence diversity compared to the PDG-B model. Given Imagen’s inherent capability in text comprehension and processing [4], our model integrates an adapted version of the Imagen framework to implement the diffusion model’s cascade [59]. Simultaneously, we simplified and optimized the accompanying denoiser neural network. Within the architecture of our denoiser, we replace traditional convolution and transposed convolution modules with multilayer bidirectional Long Short-Term Memory (Bi-LSTM) [60,61] modules. This strategic replacement avoids the risk of information distortion typically associated with convolutional operations. Consequently, our model with explicit code encapsulation proves outstanding generative capabilities, offering a more resource-efficient solution without compromising functional efficacy.

## 2. Results

### 2.1. Configurations of Our Model

Our model has considerably reduced complexity by substituting the original one-dimensional convolutional layers and residual connections with LSTM layers. In comparison to the PDG-B model, which necessitates approximately 2.6 billion (262,388,274) trainable parameters [54], our PolypeptideDesigner (PPD) model accomplishes generative tasks with merely around 1.7 billion (169,253,762) trainable parameters, achieving a considerable decrease of 35.5%. Furthermore, in terms of the capable output polypeptide length, our model nearly doubles that of the PDG-B model, increasing from 128 to 250 polypeptide residues.

### 2.2. Display of Long Polypeptide Sequences Generation

**Figure 1:**
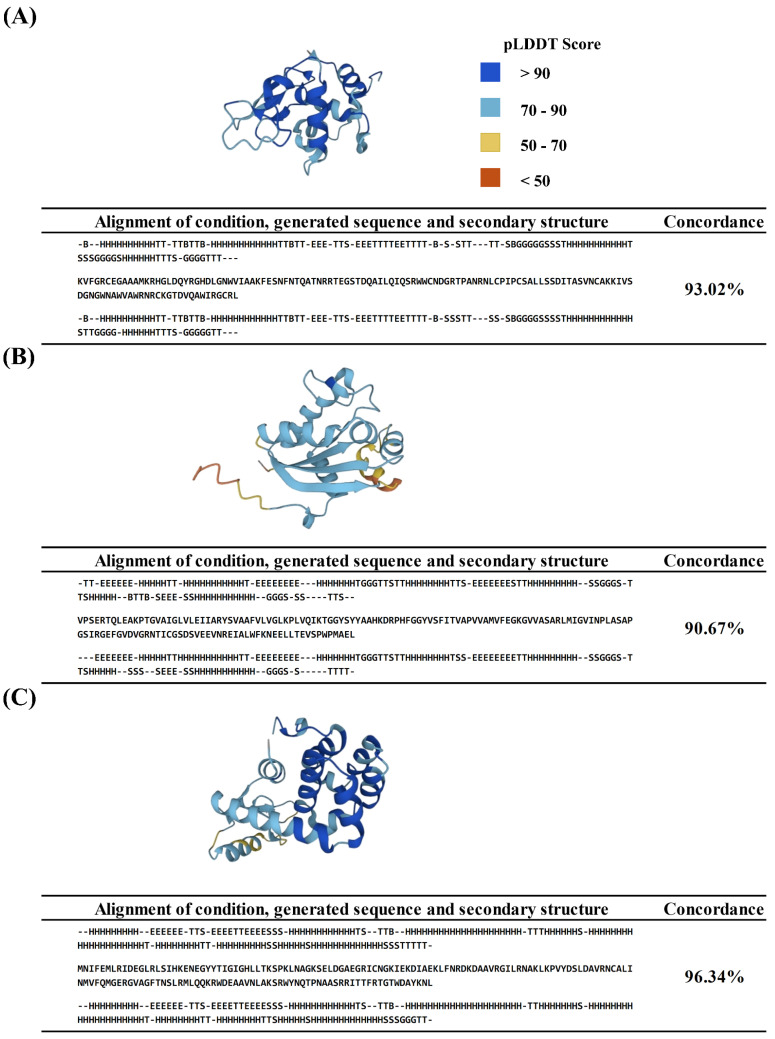
The generated results under the first 3 conditions of 6 distinct conditions from the (**A**) 4HTQ A chain (129 residues), (**B**) 1HHQ A chain (150 residues), and (**C**) 5JGR A chain (164 residues). For each condition, the folding structures of the generated sequences with the highest conformity predicted by ESMFold (top panel in each sub-figures), and the alignments of the condition sequence, generated sequence, and corresponding secondary structure sequence (bottom panel in each sub-figures). In the folding structures panel, the color of the residues represents the pLDDT Score range: dark blue for >90, light blue for 70–90, yellow for 50–70, and red for <50.

We have conducted a series of tests to evaluate the performance of our model in generating long polypeptide sequences. Considering that artificially designed secondary structure conditions may not necessarily map onto the protein space and methods to determine the physical feasibility of secondary structure conditions remain absent, we have opted to use observed secondary structures from known proteins as generation conditions for testing our model. We randomly chose 6 per-residue secondary structures from our dataset, extracted from their respective long polypeptides, to serve as the conditions for generation. The per-residue secondary structure conditions are from the A chains of 4HTQ (Figure 1A), 1HHQ (Figure 1B), 5JGR (Figure 1C), 1O2I (Figure S3D), 5E85 (Figure S3E), and 4EO9 (Figure S3F). These chosen sequences have 129, 150, 164, 223, 235, and 240 residues, respectively, with the complexity of generation progressively increasing. The sample results were processed by ESMFold and DSSP to obtain the predicted secondary structures, and subsequently, the concordance rate between these generations and the given conditions was computed. The results are detailed in Figure 1 and Figure S3, including the folding structures of the generated sequences with the highest conformity (top panel in each sub-figure of Figure 1 and Figure S3), and their alignments of the condition sequence, generated sequence, and corresponding secondary structure sequence (bottom panel in each sub-figure of Figure 1 and Figure S3). Despite the complexity of generating long polypeptide sequences, our model can generate a good concordance rate when sampled with the given conditions. As illustrated in Figure 1 and Figure S3, the best-generated sequence under each condition exhibits very high concordance rates, all exceeding 87%. Specifically, the concordance rates for (A), (B), and (C) in Figure 1 and (D) and (F) in Figure S3 surpass 90%, with (C), achieving an impressive 96.34% concordance rate.

We utilized the ESMFold Server to fold and visualize the structures of the generated sequences. As shown in top right panel of (A) in Figure 1, the color of the residues indicates the pLDDT Score range: dark blue for >90, light blue for 70–90, yellow for 50–70, and red for <50. Higher scores represent greater prediction reliability. Our model demonstrates the capability to generate high-quality sequences, with most of the generated polypeptide sequences having excellent generation quality across most structural domains, as evidenced by pLDDT scores above 70. Notably, within the (A) and (C) in Figure 1, and (D) in Figure S3, most structural domains achieved pLDDT scores above 90.

Subsequently, we conducted a homological analysis on those best-generated sequences to evaluate its novelty. Utilizing NCBI’s BLASTp tool, we searched for sequences (A)–(F) and recorded the search result with the highest e-value as the most similar homologous sequence. The results are presented in Table 1. The query cover range for retrieving homologous sequences of the (A)–(F) is between 97% and 100%, with a relatively large fluctuation in the identity with their homologous sequences, ranging from 69.46% to 90.58%. A lower identity with homologous sequences indicates a higher level of innovation in the sequences generated by the model. Among them, half of the sequences ((A), (E), and (F)) have an identity below 80%, suggesting that these sequences possess very high levels of innovation. Surprisingly, despite its length of 240 residues and the highest generation difficulty, sequence (F) exhibited excellent generation results. It achieved a high concordance rate of 90.83% with the given conditions, while maintaining a very low identity of only 69.46% with its homologous sequence. Considering that (D) has 223 residues and still differs from its homologous sequence by 21 residues, it demonstrates that the generated result incorporates a certain degree of innovative design while adhering to the conditions as much as possible (with a concordance rate of 94.62%), rather than being a mere replication of the template sequence. It is worth mentioning that although the most similar homologous sequences for E identified through BLAST are the sources of their generation conditions, all four sequences exhibit low identities and high concordance rates. Therefore, the fact that our model can de novo design long polypeptide sequences with considerable novelty has been proved. The results suggest that the model may have understood the pattern of the polypeptide sequences and was able to make some innovative substitutions of residues based on the polypeptide context and condition.

### 2.3. Performance of Short Polypeptide Sequences Generation and Improvement Comparing to PDG-B

**Figure 2:**
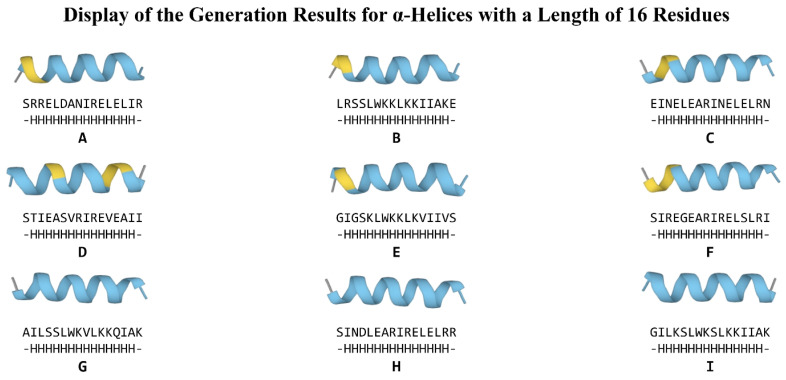
The primary structure, secondary structure, and folding structure of the best sequence generated through 50 consecutive generations under the condition of 16-residue α-helices by our PPD model. Each subplot illustrates, from top to bottom: (1) ESMFold-predicted structure of 16-residue α-helix designed by our PPD model, (2) the corresponding primary structure, and (3) per-residue secondary structure calculated via DSSP algorithm. The designed polypeptides demonstrate high structural reliability, significant sequence diversity, and strict adherence to the generative condition.

**Figure 3:**
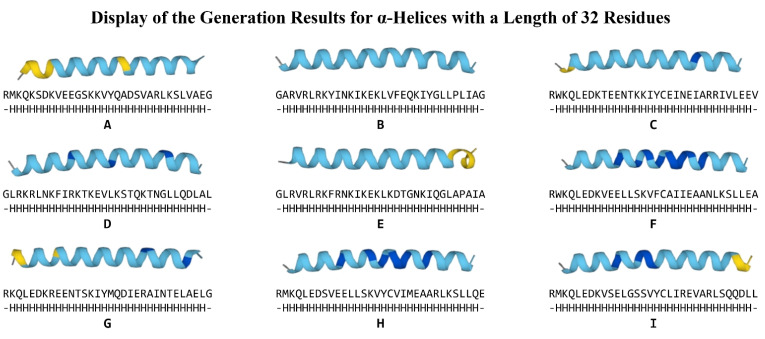
The primary structure, secondary structure, and folding structure of the best sequence generated through 50 consecutive generations under the condition of 32-residue α-helices by our PPD model.

**Figure 4:**
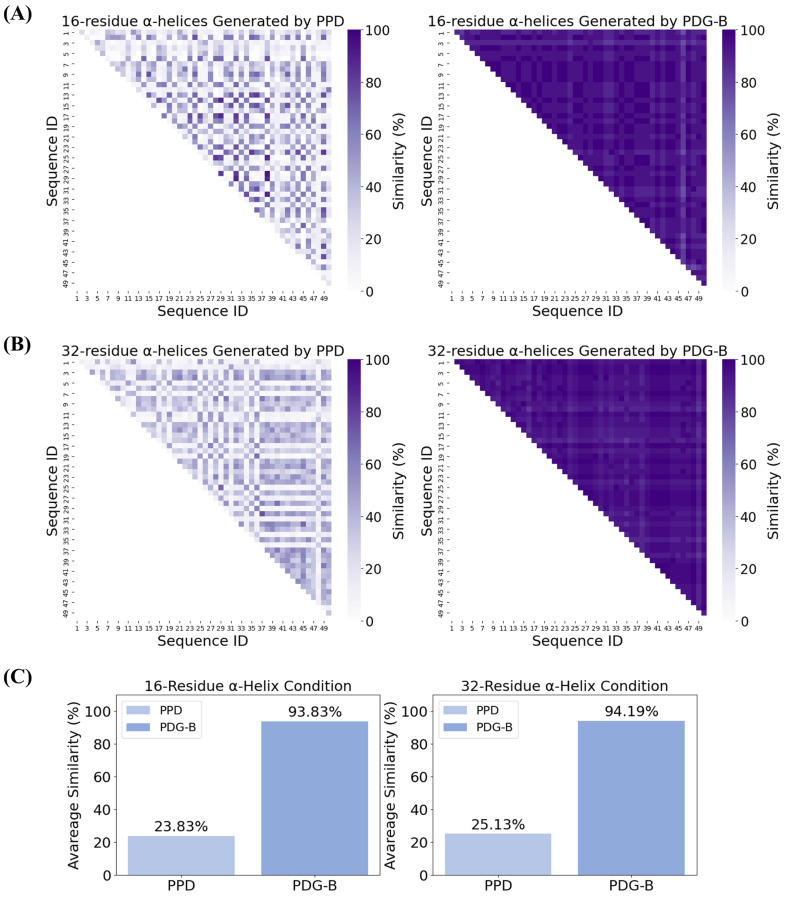
Generative diversity analysis of generated sequences under different α-helix conditions. (**A**,**B**) Heatmaps depict the pairwise similarities among the 50 sequences generated under 16- (**A**) and 32- (**B**) residue α-helix conditions, respectively. Each cell represents the similarity score between two sequences, with darker shades indicating higher similarity. (**C**) Average similarity comparison between PPD and PDG-B model under both conditions.

Due to the ubiquitous presence of α-helices in natural proteins and their relatively short-range and independent weak interactions, which imply a highly reliable physical feasibility, we employ α-helical secondary structure sequences as test conditions to evaluate and compare the PPD model and the PDG-B model. The conditional sequences consist of two components: two unstructured residues at the N-terminus and C-terminus (designated as ‘-’ in DSSP encoding), with the intervening segment being a pure α-helical region (designated as ‘H’ in DSSP encoding). We selected α-helices with lengths of 16 residues and 32 residues as generation conditions to test the PPD model and PDG-B model. With the application of protein folding technologies such as ESMFold, obtaining the folded structures of peptide sequences has become more straightforward and efficient. Consequently, the diversity and novelty of the generated polypeptide sequences are regarded as the most crucial metrics for evaluating protein generation models. We focus on assessing the generative diversity and novelty of the PPD and PDG-B models. For each experimental condition, we systematically generated sequences 50 consecutive times. The folding structures of the top nine sequences with 100% concordance rates, generated by the PPD model under both the 16- and 32-residue α-helix conditions, are presented in Figure 2 and Figure 3, respectively. Additionally, the comparative assessment between the PPD and PDG-B models are depicted in Figure 4. This figure encompasses the heatmaps illustrating the similarities among the 50 generated sequences (A,B), as well as the bar plots comparing the average similarity (C) between PPD and PDG-B.

Our analysis revealed that most of the presenting sequences generated by PPD model marked high pLDDT scores and generation quality, as Figure 2 and Figure 3 show. The generated sequences have two conformations, including straight conformations (such as H in Figure 2) and slightly curved conformations (such as D in Figure 3).

The PDG-B model tends to generate monotonous polypeptide sequences, which is confirmed by our testing on PDG-B. To visually illustrate this point, we compared upper triangular heatmaps of the similarity between the 50 sequences generated by the PPD and PDG-B models under the conditions of 16-residue (in Figure 4A) and 32-residue (in Figure 4B) α-helices, respectively. In Figure 4C, we compared the average similarity of the sequences generated by the PPD model and the PDG-B model over 50 iterations. Our PPD model consistently exhibited an average similarity below 26% across all conditions, while the PDG-B model had an average similarity exceeding 90%. The comparison reveals that the polypeptide sequences output by the PDG-B model are derived from only one polypeptide template sequence, with few residue substitutions on these templates. Compared to PDG-B, our model, maintains a high level of generative diversity while striving to adhere to the given conditions.

Next, we conducted homological analysis on the top nine sequences generated by the PPD model under each condition (corresponding to the sequences displayed in Figure 2 and Figure 3), using NCBI’s BLASTp to search against the non-redundant protein database. The results of the homological analysis are presented in Table 2 and Table 3. The sequences generated by our model demonstrate novelty, with some exhibiting very low identity to natural proteins. For instance, sequence D in Table 3 has 75% query cover and 83.33% identity with HEU0216558.1, and sequence C in Table 4 has 90% query cover and 68.97% identity with 2WQ0 A chain, among others. Our model is even capable of de novo designing polypeptide sequences that have never been observed in natural proteins, such as A, B, D, F, G, and I in Table 3. However, in Table 2, there are no sequences that are entirely absent from natural proteins. This could be attributed to the abundance of natural α-helices with 16 residues, and the possible combinations are relatively limited, thus increasing the likelihood of overlap between the generated sequences and natural protein sequences.

Given the high sequence similarity observed among the sequences generated by the PDG-B model for lengths of 16 and 32 residues, we conducted BLASTp searches only on representative sequences from these conditions. The search results are presented in Table 4 and Table 5. The results show that the sequences produced by the PDG-B model exhibit a lower novelty, sharing over 85% identity with existing sequences. Compared to the PDG-B model, our PPD model demonstrates a superior capability in generating sequences with innovative potential, enabling the de novo design of polypeptide sequences. Our research indicates that the PPD model can innovatively combine amino acid residues based on polypeptide ‘grammar’ and under the guidance of specific conditions.

## 3. Discussion

Our study introduces a diffusion-based model, named PolypeptideDesigner, designed to generate polypeptide sequences based on per-residue secondary structure conditions. Compared to the existing PDG-B model, the PPD model incorporates significant architectural improvements. Specifically, in the Denoiser module, we replace the residual-connected convolutional and deconvolutional layers with a residual-connected bidirectional multi-layer LSTM module. This design choice is motivated by the LSTM’s exceptional ability to handle long sequences and capture long-range dependencies, while avoiding the potential information distortion often associated with one-dimensional convolutions. Additionally, we have optimized and adapted the Imagen diffusion framework to better suit text generation tasks. By reducing the number of denoising iterations in the diffusion process, we significantly enhance both training and inference efficiency. Furthermore, the PPD model expands the dimensions of conditional inputs and sequence outputs, enabling the generation of sequences nearly twice as long as those produced by the PDG-B model. This advancement not only equips the PPD model with the capability to design and generate long polypeptides but also allows for the inclusion of more natural long polypeptide data in the training process. As a result, the model’s performance is enhanced, and its understanding of protein “language” is deepened. These improvements are reflected in the PPD model’s outstanding performance in generating both long and short polypeptides, demonstrating its robustness in polypeptide design.

In tests involving the design and generation of long polypeptide sequences, the polypeptides generated by PPD model demonstrate high concordance rates with the given secondary structure conditions. The structural quality of the generated sequences, as evaluated by ESMFold, further underscores the model’s effectiveness. Most generated sequences exhibited high pLDDT scores (>70), with many structural domains achieving scores above 90, indicating reliable structural predictions. This suggests that the PPD model not only adheres to secondary structure conditions but also produces sequences with physically feasible and stable folds. Homology analysis revealed that the generated sequences exhibit a certain degree of novelty. For example, sequences with 240 residues achieved a low identity of 69.46% with their closest natural homologs, demonstrating the model’s ability to innovate. At the same time, some generated sequences showed more conservative designs, with homology around 90% to natural proteins. Taken together, these results—spanning both high and low homology—suggest that the PPD model can flexibly adopt either conservative or innovative design strategies based on the context of the structural information.

In the generation of short polypeptide sequences, the PPD model also excels. Using 16- and 32-residue α-helical structures as conditions, we compared the performance of the PPD and PDG-B models. The PPD model significantly outperforms the PDG-B model in terms of sequence diversity, with an average similarity below 26%, compared to over 90% for the PDG-B model. The short polypeptide sequences generated by the PPD model exhibit high concordance rates with the target conditions, high-quality structural domains, and diverse conformations (e.g., straight and curved α-helical structures). Moreover, homology analysis reveals that some of the short polypeptide sequences generated by the PPD model have extremely low homology with natural sequences, and some are entirely novel, not observed in nature. This performance can be attributed to the optimized model architecture and the expanded training dataset.

The results from both long and short polypeptide tests demonstrate the PPD model’s robust capability in polypeptide design and innovation, generating high-quality sequences that align well with design objectives. This balance between adherence to conditions and sequence novelty indicates that the PPD model has effectively learned the pattern of polypeptide sequences.

Our study demonstrates the powerful potential of diffusion models, highlighting their broad applicability in protein design. The PPD model is inherently well suited for tasks such as optimizing known polypeptide biomaterials or drugs. By leveraging known structural information, the model can design potential alternative peptide chains, thereby exploring and enhancing material or drug performance. This capability shortens the development cycle for materials and drug design. The PPD model not only surpasses existing methods in generation efficiency but also exhibits remarkable advantages in sequence diversity and innovation. It provides a novel tool and methodology for protein design, effectively addressing the gap in long peptide design.

However, it is important to acknowledge that our study still has certain limitations. When generating long peptides, the PPD model produces some sequences with homology around 90%, which, according to the principles of classical homology modeling [62,63], is considered relatively high. The model’s protein sequence design capability is inherently constrained by the fundamental limitations of diffusion models, which may lead to conservative generation strategies in certain cases. It is worth noting that the baseline method, PDG-B, also suffers from this issue, and the results show that it performs even worse than our PPD model. Subsequent improvements include but are not limited to: (1) redesigning the model’s architecture, (2) developing a novel homology-aware loss function with adaptive regularization, and (3) integrating multimodal biological and physical information.

In addition, both the PPD and PDG-B models exhibit suboptimal performance when generating short polypeptides dominated by β-sheets (i.e., when β-sheets account for more than 60% of the total length). This limitation may stem from insufficient training data and inherent physical infeasibility. Regarding data bias, as illustrated in Figures S1 and S2, polypeptides with β-sheets constituting over 50% of their total length are scarce in our database, with the majority falling below this threshold. Generation models are data-driven, and their performance is directly linked to the amount of data. From a physical perspective, short β-sheet-dominated polypeptides are instable. The infeasibility of such generation conditions prevents the model from successfully mapping the conditions to the polypeptide sequence space. We will continue to address the current limitations, such as the high homology of some generated sequences and the suboptimal performance in β-sheet generation.

## 4. Materials and Methods

### 4.1. Overview of Our Study

**Figure 5:**
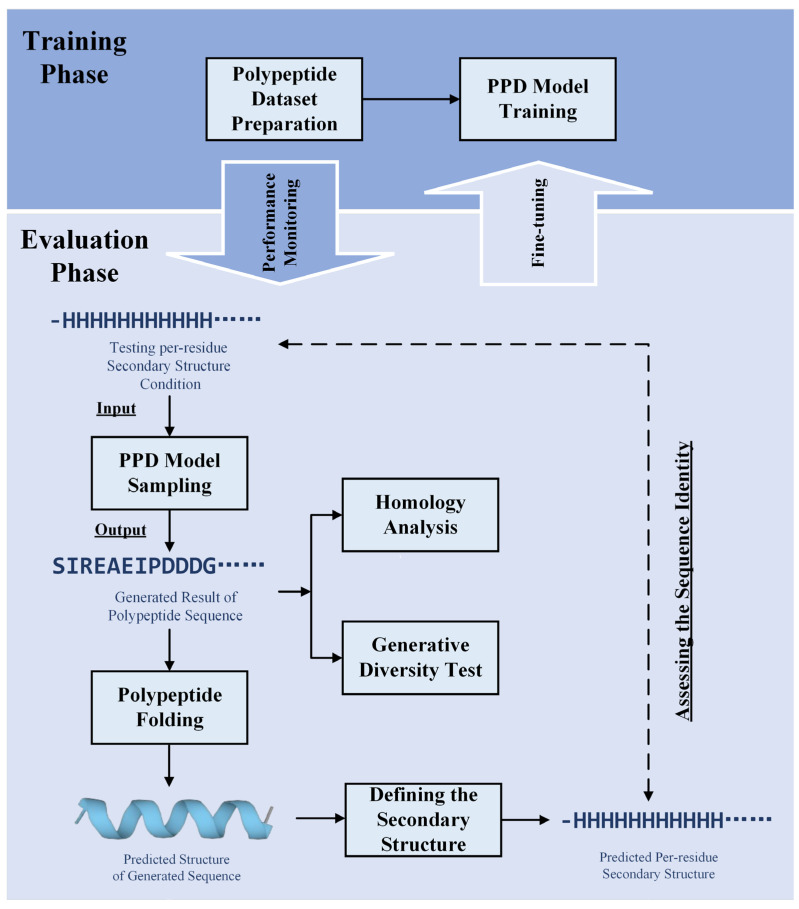
Overview of our study. Our research comprises two main phases: model training and evaluation. In the training phase, we prepare a polypeptide dataset and train the PPD model. During evaluation, we input per-residue secondary structure into the trained PPD model to generate amino acid sequences. These generated sequences undergo analysis to assess model performance, including secondary structure prediction with ESMFold and DSSP, homology analysis with NCBI’s BLASTp for novelty, and diversity tests among generated sequences. We measure novelty by comparing generated sequences to natural ones and diversity by assessing similarity among sequences generated under the same conditions. Model fine-tuning and comprehensive evaluations have led to the development of a robust polypeptide design model.

Figure 5 displays the overview of our study. As Figure 5 shows, our research comprises two primary phases: model training and evaluation. In the training phase, we prepare a comprehensive polypeptide dataset and train the PPD model. During the evaluation phase, per-residue secondary structure sequences are input into the trained PPD model to generate corresponding amino acid sequences. The generated sequences are then rigorously analyzed to assess the model’s performance. This analysis includes secondary structure prediction using ESMFold and DSSP [64], homology analysis via NCBI’s BLASTp to evaluate novelty, and diversity testing among the generated sequences. Generation novelty was quantified by comparing the generated sequences to natural polypeptide sequences (query cover and identity of natural polypeptide sequence with the highest e-value from a BLASTp search), while generation diversity was assessed by measuring the similarity among sequences produced under identical conditions. Through iterative fine-tuning and comprehensive evaluations, we have developed a robust and reliable model for polypeptide design.

### 4.2. Data Processing and Encodings

We have endeavored to download as many recent protein structures stored in the macromolecular Crystallographic Information File (mmcif) as feasible from the PDB website. Utilizing the Biopython library [65], a renowned Python tool for bioinformatics research, we extracted all polypeptide chains from each protein structure archived within these files. A total of 501,534 polypeptide chains were extracted from all protein structures we collected. After data deduplicating and cleaning, we then employed the DSSP method to define the secondary structures for each residue within the extracted polypeptide chains, which were then concatenated to form per-residue secondary structure sequences. A total of 40,848 polypeptide sequences were compiled within our database, spanning sequence lengths of up to 400 residues. For the ensuing training phase, we selectively utilized 28,141 sequences, all of which were constrained to a length of 250 residues or fewer.

Additionally, we computed various sequence features, including sequence lengths and the proportions of different secondary structures. A statistical analysis, including the distribution of sequence lengths in the dataset spanning from 1 to 250 residues, the distribution of secondary structure elements in the dataset, the distribution of amino acid residues, and the joint distribution of proportional contents for a specific pair of secondary structures, was performed on our database to ensure the absence of data bias, and the results are presented in Figures S1 and S2.

The collected polypeptide sequences and their corresponding secondary structure sequences are encoded from strings into arrays according to specific encoding rules. The encoding rules for the 8 secondary structures and 20 amino acids are, respectively, presented in Table 6 and Table S1. Subsequently, these encoded arrays undergo normalization and are padded with zeros at the end to ensure a uniform length of 250. The processed data are then managed and labeled by a subclass inherited from PyTorch’s Dataset and eventually partitioned into batches and converted into tensors by PyTorch’s DataLoader [66].

### 4.3. Diffusion Framework and Neural Network of Denoiser

**Figure 6:**
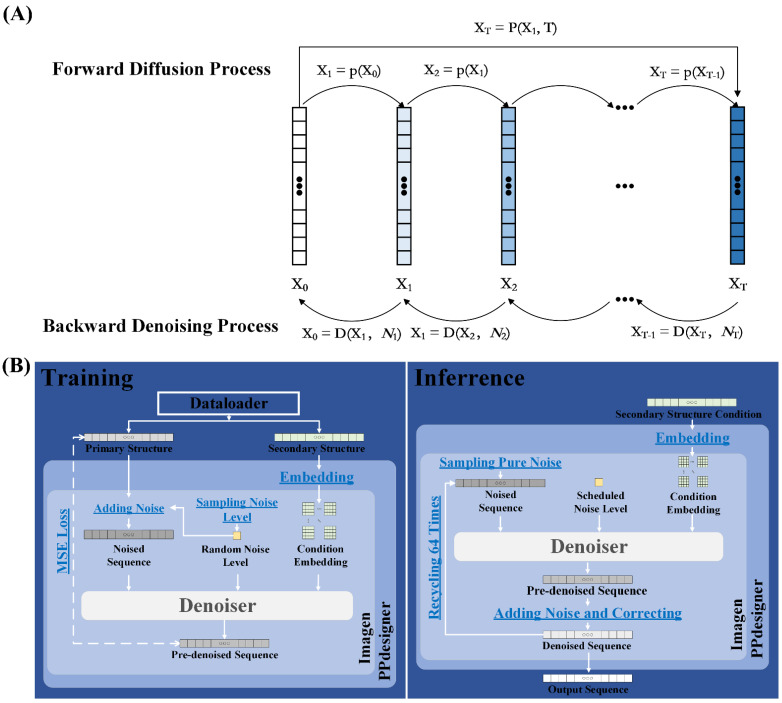
The figure illustrates the fundamental principles underlying diffusion models, as well as the implementation and workflow of our specific model. (**A**) The mechanism of the diffusion model consists of two fundamental processes: a forward diffusion process and a reverse denoising process. Forward diffusion gradually adds noise, and reverse denoising progressively removes noise to generate data. (**B**) In our model, training and inference are independently implemented. Our model utilizes three custom subclasses, namely PPdesigner, Imagen, and Denoiser, which are inherited from nn.Module, the base class of PyTorch. Each class has distinct roles in training and inference.

The diffusion model’s mechanism, depicted in Figure 6A, is fundamentally comprising two primary processes: a forward diffusion process and a reverse denoising process, as demonstrated in the original DDPM paper [1].

The forward diffusion process iteratively adds Gaussian noise to the initial polypeptide sequence over T timesteps, gradually transforming it into Gaussian-like noise. This Markov process, where each step depends only on the current state, can be described by Equation (1):

(1) $$X_{i}=p\left(X_{i-1}\right)$$

Through mathematical derivation, multiple noise additions can be simplified to a single weighted Gaussian noise operation (Equation (2)):

(2) $$X_{T}=P\left(X_{1},T\right)$$

where $X_{T}$ and $X_{1}$ represent the final and initial sequences, respectively. This formulation reduces computational complexity while effectively augmenting training data across diffusion timesteps.

The reverse denoising process employs a trained denoiser to reconstruct the target sequence from noise, as described by Equation (3):

(3) $$X_{i-1}=D\left(X_{i},N_{i}\right)$$

where $X_{i}$ and $X_{i-1}$ represent current and previous denoising states, and $N_{i}$ denotes the scheduled noise level. This iterative process enables precise sequence generation while maintaining computational efficiency.

Our model employs Mean Squared Error (*MSE*) as the loss function (Equation (4)):

(4) $${Loss}_{MSE}=\frac{1}{N}\sum_{i=1}^{N}{\left({\hat{Y}}_{i}-Y_{i}\right)}^{2}$$

where N is the total number of samples (batch size × sequence length), and ${\hat{Y}}_{i}$ and $Y_{i}$ represent the predicted and true values for the i-th residue, respectively. This metric quantifies reconstruction accuracy, reflecting the denoiser’s sequence understanding and denoising capability.

The implementation of our model is powered by PyTorch [66] and TensorFlow [67]. Moreover, Imagen is deployed in our model to implement an improved diffusion cascade. Figure 6B is a schematic diagram illustrating our diffusion framework. Our model primarily implements the diffusion process through the nested and encapsulated structure of three custom child classes of nn.Module (base class within the PyTorch library): PPdesigner, Imagen, and Denoiser. These three classes undertake distinct but relatively independent responsibilities during both training and inference phases. To optimize computational efficiency, we revise and simplify the Imagen block. Specifically, we randomly sample the diffusion timestep for adding noise and denoising just once per training batch, applying it uniformly to all sequences within that batch, rather than sampling individually for each sequence within that training batch.

As illustrated in Figure 6B, the training and inference phases are implemented independently and, respectively, in our model. During the training phase, the primary and secondary structure sequences, dispensed by the Dataloader, are passed to PPdesigner. PPdesigner is responsible for embedding the secondary structure conditional sequences and subsequently forwarding them along with the primary structure sequences to Imagen. Imagen then samples random noise levels to control the intensity of noise added to the primary structure sequences. The noised primary structure sequences, the sampled noise levels, and the embedded secondary structure conditions are jointly fed into Denoiser for denoising, producing a pre-denoised sequence. Finally, Imagen computes the model’s loss function and optimizes the parameters.

During the inference phase, a user-defined secondary structure conditional sequence is transmitted to PPdesigner, where it is embedded and subsequently passed to Imagen. Imagen then samples a pure Gaussian noise sequence, which, along with the planned corresponding noise levels and the conditional embeddings, is input to Denoiser for denoising, outputting a pre-denoised sequence. Imagen further processes this pre-denoised sequence by adding additional Gaussian noise to enhance creativity and applying corrections to ensure sequence reliability. This iterative process of denoising and correction is repeated 64 times, progressively refining the sequence, ultimately generating the desired output.

**Figure 7:**
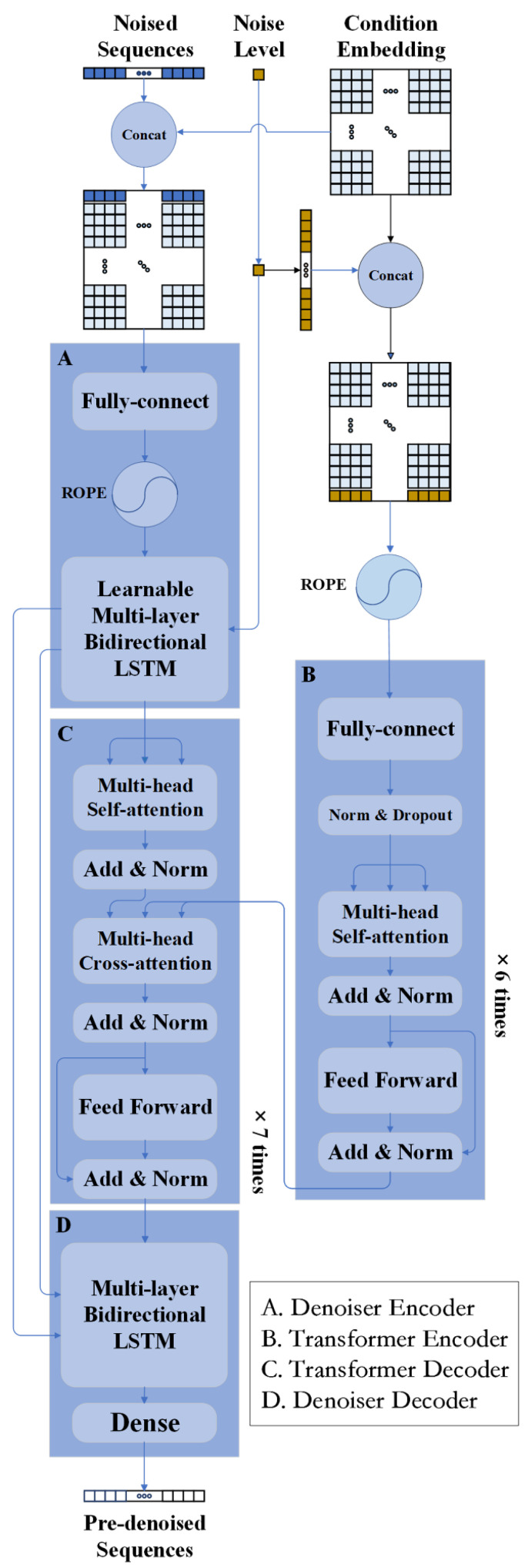
Architecture of the Denoiser in our model. The Denoiser is a seq2seq neural network model, receiving noise sequences ([batch size, 250]), condition embeddings ([batch size, 250, 256]), and noise level (float), and outputting denoised sequences ([batch size, 250]). The Denoiser mainly comprises four components: Denoiser Encoder (A), Transformer Encoder (B), Transformer Decoder (C), and Denoiser Decoder (D). The Denoiser Encoder and Denoiser Decoder are additionally connected by passing the hidden state and cell state tensors in LSTM. Several advanced neural network techniques include bidirectional multi-layer LSTM, multi-head attention, and ROPE are deployed in our model.

To better adapt the diffusion model for the task of text generation, we have designed the LSTM/attention-based Denoiser, serving as a replacement for the U-Net denoiser originally paired with Imagen. Our devised architecture of the Denoiser is illustrated in Figure 7. As Figure 7 shows, the Denoiser neural network is a sequence-to-sequence (seq2seq) model, receiving 250 length noised sequences (2 dimensions tensor with shape [batch size, sequence length]—[batch size, 250]) as noise input, secondary structure condition embeddings (3 dimensions tensor with shape [batch size, sequence length, dimension of condition embedding]—[batch size, 250, 256]) as condition input, and the noise level (a float) as noise level input. Subsequently, it outputs 250 length denoised sequences (2 dimensions tensor with shape [batch size, sequence length]—[batch size, 250]). Its structure primarily consists of 4 components: a Denoiser Encoder for feature extraction from noised sequences, a Transformer Encoder for condition feature extraction, a Transformer Encoder for fusion between the latent feature and condition feature, and a Denoiser Decoder for recovering polypeptide sequences from the latent feature. We incorporate several advanced neural network techniques, notably including bidirectional LSTM in both the encoder and decoder, multi-head attention (cross-attention and self-attention) within transformer blocks, and learnable Rotary Position Embedding (RoPE) [68] to incorporate sequence-relative position information. It is noteworthy that the noise level, after being repeated a fixed number of times and padded into tensors (3 dimensions tensor with shape [number of LSTM layers × number of LSTM directions, batch size, LSTM’s hidden size]—[12, batch size, 512]), serve as the initial cell states in learnable multi-layer bidirectional LSTM of the Denoiser Encoder. Apart from that, a tensor with learnable parameters (3 dimensions tensor with shape [number of LSTM layers × number of LSTM directions, batch size, LSTM’s cell size]—[12, batch size, 512]) serves as the initial hidden state in the learnable multi-layer bidirectional LSTM of the Denoiser Encoder. Then, the output hidden and cell states from the learnable multi-layer bidirectional LSTM in the Denoiser Encoder are transmitted to the multi-layer bidirectional LSTM in the Denoiser Decoder, functioning as their initial hidden and cell states. The design of directly connecting LSTM blocks in the Denoiser Encoder to LSTM blocks in the Denoiser Decoder through the transmission of hidden states and cell states is inspired by the residual connections in the classic U-Net architecture [58]. This allows deep layers of the network to still receive information from superficial layers, while the forms of hidden states and cell states preserve the holistic features of long sequences from shallow networks, therefore exhibiting better compatibility with languages and long sequences. The experimental outcomes suggest that this architecture of the Denoiser we have designed possesses potential for application in other generalized seq2seq or NLP tasks. The parameters and hyperparameters of our model, along with Adam optimizer [69], are detailed in Table S2.

### 4.4. Enhancing Robustness and Generative Diversity Through Dropout Regularization and Classifier-Free Guidance

We have made efforts to enhance the robustness and generative diversity of our model. In our model, we deploy dropout regularization on the entire text embeddings to enhance the model’s understanding of polypeptide sequence context and reduce its reliance on individual conditions [70,71]. In addition, the classifier-free guidance (CFG) technique has been integrated into our model, enabling it to generate sequences conditionally without the guidance of an explicit classifier [72]. This approach circumvents the additional computational overload associated with classifier training and avoids the risk of the model attempting to deceive the classifier. During the training phase, joint training of the model is achieved through dropout along the sequence length dimension of the conditional text embeddings. Therefore, this allows the model to generate sequences both conditionally, guided by specific constraints, and, unconditionally, following the overall distribution of the polypeptide dataset, based on given probability parameters. In the generation phase, the model synthesizes the results from both conditional and unconditional generations by assigning weights to each, calculated as Equation (5):

(5) $$X_{CFG}=X_{unconditional}+W\left(X_{conditional}-X_{unconditional}\right)$$

where $X_{unconditional}$ denotes the output generated without conditional guidance, $X_{conditional}$ denotes the output generated under conditional constraints, and W denotes the condition scale, a parameter that regulates the mixture between the conditional and unconditional generations. In our model, the condition scale W is set to 1, which signifies that the model adopts the results from fully conditional generation.

### 4.5. Training Details

For model training, the Adam optimizer was employed, with the learning rate progressively decreasing from 10 to -4 to 10 to -7. The training dataset was incrementally expanded, starting from 25% and then scaling up to 65%, ultimately increasing to 95% of the total dataset.

We adopt a pretraining strategy aimed at enhancing training efficiency. Initially, we randomize all model parameters by PyTorch’s default and allow backpropagation across all layers, training the model on a smaller subset of the data which comprises from 25% to 65% of the total dataset. This phase continues until the model’s loss decreases from around 0.7 to approximately 0.25. Subsequently, we augment the training set to 95% of the total dataset and proceed to fine-tune the model. During this fine-tuning phase, we fixed part of the model parameters, permitting backpropagation only for the LSTM layers within the encoder/decoder, the decoder layers in the Transformer, and the final dense layer responsible for output. This training strategy effectively balances both training efficiency and model reliability. We rigorously monitor and save each set of model parameters, subjecting them to generation efficacy tests. Early stopping is implemented once the generation performance begins to deteriorate, ultimately allowing us to select the parameter set that yields the best generation results.

For utilization and further study, two sets of parameters are provided. One set encompasses the optimal performance PPD parameters, while the other provides early-stage training parameters for transfer learning.

### 4.6. Protein Folding, Secondary Structure Parsing and Structure Visualizing

We utilized the ESMFold server by uploading generating sequences and then downloading protein structure files via its web interface. For those interested, the encapsulation code pertaining to the ESMFold web interface can be found within our codebase. The predicted protein structures are often evaluated using the pLDDT metric, which provides a quantitative measure of the local quality of the prediction at residue-level, with higher scores indicating higher confidence in the prediction.

The folding structures predicted from ESMFold in PDB file format are delivered to PDBParser, a module in Biopython library, and then we parse the secondary structure by DSSP through the interface in Biopython. We accept the per-residue accuracy $A_{pr}$ referred from PDG-B model’s essay to quantify to what extent the generated sequences according with constraint conditions [54], as Equation (6) shows

(6) $$A_{pr}=\frac{n}{N}$$

where n represents the number of residues at corresponding positions in the sequence that satisfy the secondary structure constraints, and N represents the total number of amino acid residues in the sequence.

The predicted polypeptide structures in PDB file format are visualized by ESMFold server.

### 4.7. Homology Analysis for Generation Novelty and Generation Diversity Test of Sample Polypeptide Sequences

The homology assessment of the sample sequences was conducted through the application of the BLASTp algorithm, a specialized tool for protein-to-protein sequence alignment, in conjunction with the National Center for Biotechnology Information’s (NCBI) non-redundant (nr) protein sequence database. This methodology ensured a comprehensive and reliable analysis of protein sequence similarity and homology, thereby facilitating a clear elucidation of the innovation level inherent in the designed polypeptide sequence. We select the natural polypeptide sequence with the highest e-value from a BLASTp search to quantify the level of homology, considering both the query cover and identity of the natural sequence. By using the powerful alignment tool—BLASTp and the abundant sequence data available in the nr database, we can assess the generation novelty.

To quantify the diversity of polypeptide chains generated by generative models, we propose the use of the average similarity proportion to measure the diversity of the generations, which can be calculated using Equation (7):

(7) $$S_{avg}=\frac{\sum_{i=1}^{N_{pair}}S_{i}}{N_{pair}}$$

In Equation (7), $S_{avg}$ represents the average similarity across all pairs. The numerator, $\sum_{i=1}^{N_{pair}}S_{i}$, is the sum of individual similarity $S_{i}$, where each $S_{i}$ measures the similarity between a specific pair of generated sequences. The denominator, $N_{pair}$, denotes the total number of pairs for which similarity have been calculated.

We take the following measure to evaluate the generating performance: the generative model is tasked with consecutively generating 50 polypeptide chains under the same secondary structure condition. Subsequently, we perform pairwise comparisons among the 50 generated primary structure sequences of the polypeptide chains, counting the number of identical residues at corresponding positions and calculating the percentage of these identical residues relative to the total sequence length as the similarity. Then, to visually assess the diversity generated by the model, we construct a heatmap depicting the similarities among all 50 sequences. Lastly, the average similarity among the sequences is computed, excluding similarity data from the diagonal and lower triangle of the matrix during the calculation.

### 4.8. Hardware and Software Configuration

For the purpose of model training, we utilized an NVIDIA Tesla V100 GPU equipped with 32 GB of VRAM. Additionally, our software setup comprising Python version 3.8.12, Biopython version 1.82, PyTorch version 2.0.1, TensorFlow version 2.11.0, and CUDA version 12.2.

## 5. Conclusions

We propose a conditional text generation model based on diffusion models for the de novo design of long polypeptide sequences, paired by a lightweight denoising neural network utilizing bidirectional multi-layer LSTM and self/cross attention mechanisms. This model can generate protein sequences ranging from 1 to 250 residues under the guidance of per-residue-level secondary structure conditions. A series of evaluations were conducted on both our proposed model, termed PPD, and the PDG-B model. The results demonstrate that, while adhering closely to the generation conditions, the PPD model can generate polypeptide sequences that do not exist in natural proteins (see BLASTp results in Table 4) or novelly substituting and modifying residues within existing polypeptide sequences (see BLASTp results in Table 1, Table 2 and Table 3). The sequences generated by our PPD model have exceptional diversity and innovation, and most of them possess high reliability when folded by ESMFold. In comparison, although the PDG-B model outperforms PPD model in terms of adhering to the conditions, this is attributed to the lack of generation diversity. PDG-B only generates highly similar polypeptide sequences. The performance of the PDG-B model suggests that, in some cases, it may memorize and repeat template sequences that meet the conditions, rather than understand the intrinsic relationships between secondary and primary structures. In addition, we have openly shared the details of our PPD model, including relevant parameters, hyperparameters, and training methodologies, for reproducing or transfer learning in related fields.

Our model facilitates the design of novel polypeptide materials and the modification and enhancement of physicochemical properties in existing ones. By innovatively substituting residues or creating entirely new combinations of amino acid residues within the context of preserving the secondary structural domains of native polypeptides, we aim to explore new possibilities. Given that the novel sequences share the same secondary structures as their natural counterparts, they may exhibit functional similarities; however, differences in residue composition could lead to distinct material properties. Upon synthesis and testing, these artificially designed polypeptide sequences are likely to outperform. Our model substantially accelerates the research and development cycle for these new materials.

With the progression of protein structure folding technologies, we are now better equipped to predict protein structures and derive their corresponding conformations from artificially designed polypeptide sequences. In future research, the utilization of these technologies will enable us to enrich protein databases and training sets, transitioning from only use of natural polypeptides to concurrent use of both artificially designed and natural polypeptides for model training, thereby enhancing the performance of our models. These artificially designed sequences can originate from natural sequences, generated automatically by truncating natural sequences, adding, deleting, or substituting specific residues, like data augmentation techniques in deep learning. Subsequently, those sequences are folded by ESMFold, AlphaFold2, AlphaFold 3, and so on, to obtain their folding structure. The employment of such data-augmented datasets may significantly bolster the robustness and generalization capability of the models, mitigating the risk of overfitting as well.

In other future endeavors, we intend to delve deeper and more comprehensively into the utilization of polypeptide data and features. By integrating multiple polypeptide features, we aim to train a diffusion model that has a more intricate architecture with a greater number of parameters, capable of accepting multimodal conditions. This approach is designed to enhance the model’s understanding of polypeptide structures. Those features may encompass contact maps and distance matrices of polypeptide structures, the cellular localization of polypeptides, results from multiple sequence alignments of polypeptides, as well as functional descriptions of polypeptides, among other multimodal features. Ultimately, our goal is to construct a universal multimodal-condition-to-protein design generation model based on the diffusion framework.

We will also endeavor to apply transfer learning and fine-tuning to our PPD model, aiming to adapt it to various other tasks and thereby test its potential for broader applications. For instance, the PPD model can be fine-tuned and transferred to predict the deleteriousness of polypeptide sequence mutations. This involves inheriting the parameters of the original PPD model, adding extra linear layers and modules to reshape the size of the output tensors, and training with certain parameters frozen based on the pre-trained weights, to meet the specific requirements of the task. Given the potential deep connections between protein design and mutation deleteriousness, along with the abundance of successful case studies in transfer learning, the application of the PPD model through transfer learning to predict mutation deleteriousness holds considerable theoretical feasibility.

## Figures and Tables

**Table 1:** Homological analysis results of generated sequence with highest conformity under (A)–(F) condition.

Index	Length	Condition Source	Homological Analysis Result
A	129	4HTQ A chain	100% query cover; 89.92% identical with PDB: 1FLW A chain
B	150	1HHQ A chain	97% query cover; 76.71% identical with PDB: 1NDK A chain
C	164	5JGR A chain	100% query cover; 89.02% identical with PDB: 3CDT A chain
D	223	1O2I A chain	100% query cover; 90.58% identical with NP_001107199.1
E	235	5E85 A chain	100% query cover; 70.64% identical with PDB: 5E85 A chain
F	240	4EO9 A chain	99% query cover; 69.46% identical with WP_010908901.1

**Table 2:** Homological analysis results of 16-residue α-helices sampled by PPD in Figure 2.

Index	Concordance	Homological Analysis Result
A	100.00%	75% query cover; 91.67% identical with WP_309399408.1
B	100.00%	81% query cover; 100.00% identical with PDB: 5MMK A chain
C	100.00%	81% query cover; 92.31% identical with MDE5972714.1
D	100.00%	75% query cover; 83.33% identical with HEU0216558.1
E	100.00%	75% query cover; 91.67% identical with MEI6755390.1
F	100.00%	100% query cover; 87.50% identical with PDB: 1S9Z A chain
G	100.00%	93% query cover; 86.67% identical with PDB: 5MMK A chain
H	100.00%	93% query cover; 86.67% identical with PDB: 1S9Z A chain
I	100.00%	100% query cover; 87.50% identical with PDB: 5MMK A chain

**Table 3:** Homological analysis results of 32-residue α-helices sampled by PPD in Figure 3.

Index	Concordance	Homological Analysis Result
A	100.00%	No significant similarity found
B	100.00%	No significant similarity found
C	100.00%	90% query cover; 68.97% identical with PDB: 2WQ0 A chain
D	100.00%	No significant similarity found
E	100.00%	100% query cover; 78.12% identical with PDB: 1LYP A chain
F	100.00%	No significant similarity found
G	100.00%	No significant similarity found
H	100.00%	100% query cover; 71.88% identical with PDB: 2WPY A chain
I	100.00%	No significant similarity found

**Table 4:** Homological analysis results of a representative 16-residue α-helix sampled by PGD-B.

Concordance	Homological Analysis Result	Generated Sequence
100.00%	87% query cover; 100.00% identical with PDB: 1S9Z A chain	SIRELEARIRELELIT

**Table 5:** Homological analysis results of a representative 32-residue α-helix sampled by PGD-B.

Concordance	Homological Analysis Result	Generated Sequence
100.00%	100% query cover; 93.75% identical with XP_051707010.2	GKRKRLRKFRNKIKEK LKKIGQKIQGLLNKLA

**Table 6:** The table lists the DSSP secondary structure codes, their corresponding specific secondary structures, and the encodings used to represent them.

DSSP Code	Corresponding Secondary Structure	Encoding
H	α-helix	1
E	Parallel or antiparallel β-sheet	2
-	None	3
T	Hydrogen bonded turn	4
S	Bend	5
G	3_10_ helix	6
B	β bridge	7
I	π helix	8

## Data Availability

The PPD model code, training datasets and model parameters can be accessed through GitHub: https://github.com/daedaluser/PPD.git (accessed on 1 January 2025).

## Supplementary Materials

The following supporting information can be downloaded at: https://www.mdpi.com/article/10.3390/molecules30051116/s1, Figure S1: Amino acid distribution in our database; Figure S2: Secondary structure distribution in our database; Figure S3: Long polypeptide generation performance under other three conditions; Table S1: Encoding scheme for amino acid; Table S2: Model parameters and hyperparameter configurations.
