# Supplementary material for: De Novo Design of Large Polypeptides Using a Lightweight Diffusion Model Integrating LSTM and Attention Mechanism Under Per-Residue Secondary Structure Constraints
Source: Molecules. 2025 Feb 28;30(5):1116. doi: 10.3390/molecules30051116 (PMC11902264; doi:10.3390/molecules30051116)
Supplement: Supplementary file 1 [file molecules-30-01116-s001.zip › molecules-3427286-supplementary.pdf]

Supplementary Materials

De Novo Design of Large Polypeptides Using a Lightweight Diffusion Model Integrating LSTM and Attention Mechanism under Per-Residue Secondary Structure Constraints

Sisheng Liao, Gang Xu, Li Jin, & Jianpeng Ma*

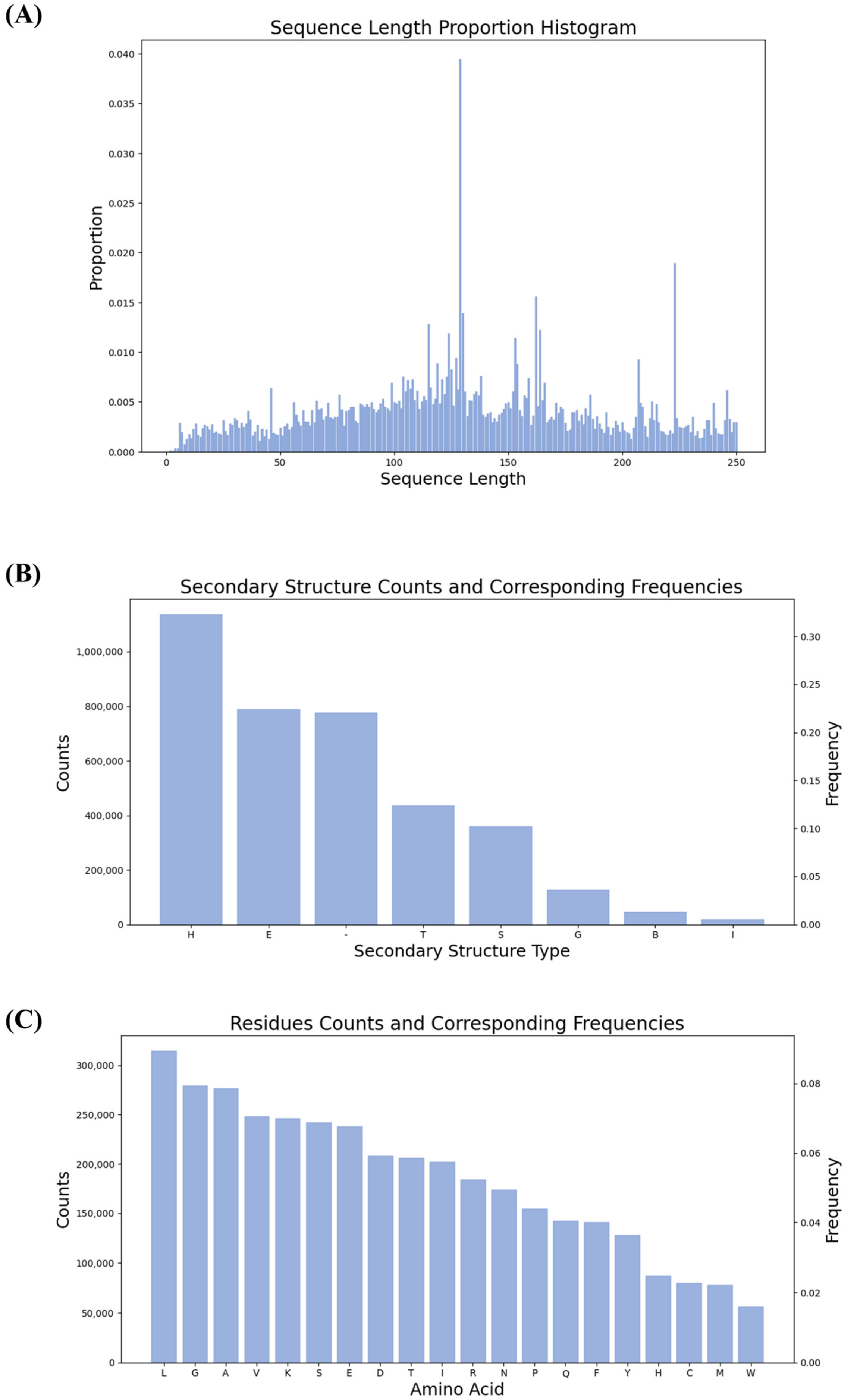

Figure S1. The results of statistical analysis conducted on our dataset. **(A)** Distribution of sequence lengths in the dataset, spanning from 1 to 250 residues. **(B)** Distribution of secondary structure elements in the dataset. **(C)** Distribution of amino acid residues.

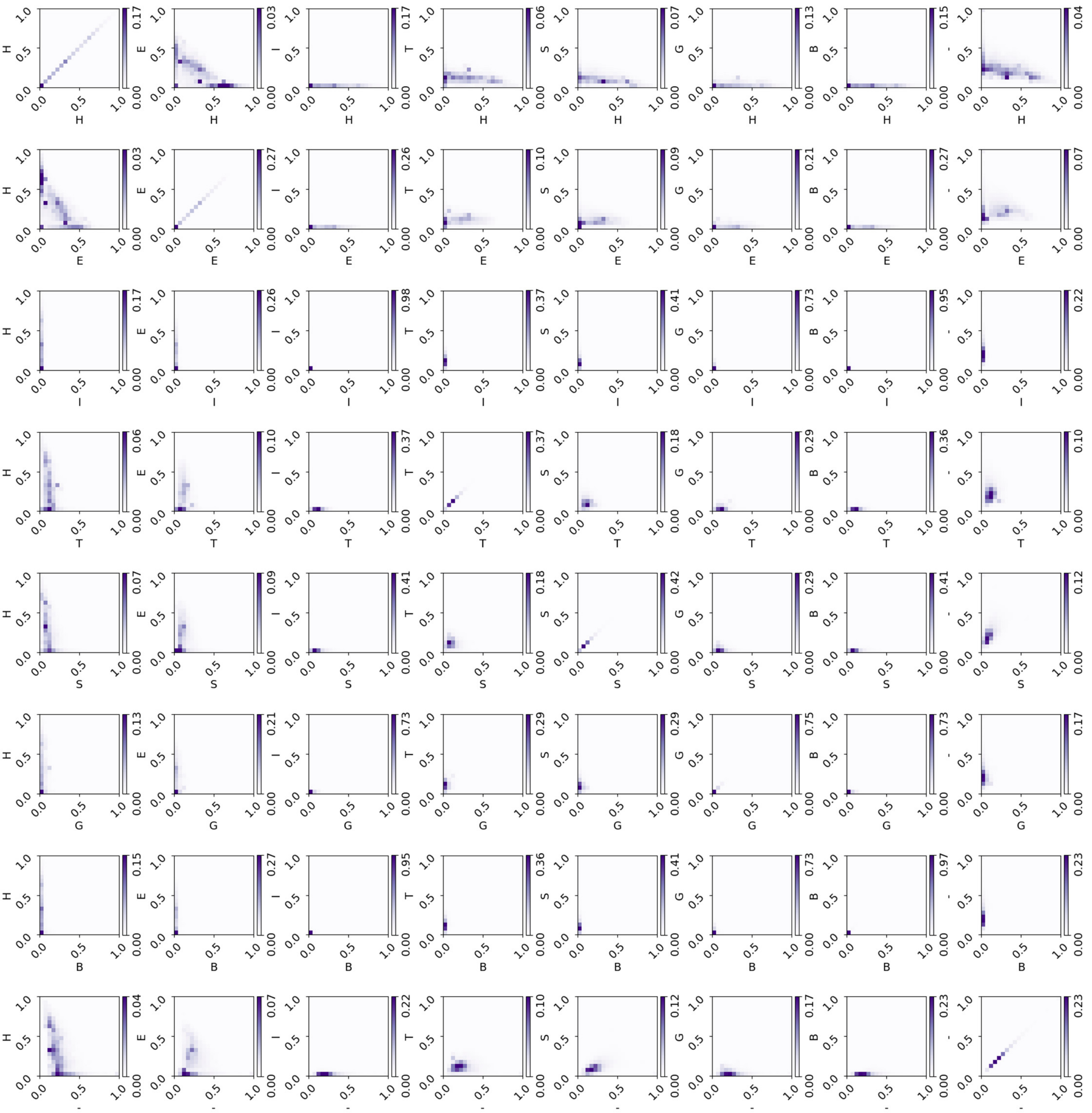

Figure S2. The figure presents a comprehensive 2D histograms depicting the co-occurrence probability distribution of pairwise secondary structure composition in our polypeptide database. The 8×8 array of heatmaps represents all possible combinations of 8 secondary structure types, where each heatmap visualizes the joint distribution of proportional contents for a specific pair of secondary structures across all polypeptide chains. Each heatmap's x- and y-axes represent the fractional content (0-1) of two distinct secondary structure types relative to the total peptide length, with color intensity indicating the probability density of observing composition combinations. The data were generated by computing the secondary structure composition for each polypeptide using DSSP encoding rules (Table 1).

(D)

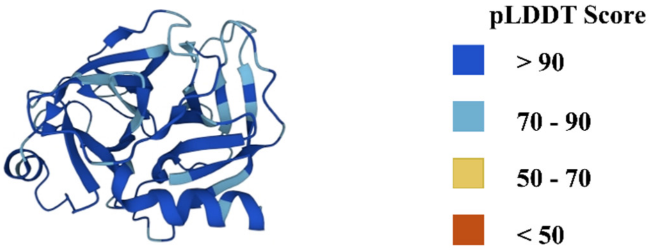

Alignment of condition, generated sequence and secondary structure	Concordance
-BS-EE--TTSSTTEEEEEESSSEEEEEEEEEETTEEEE-GGG--SS-EEEES-SSTTS--S--EEEEEEEEEE-TT--TTT-TT--EEEEESS----BTTB<br>---B--SS---TT-EEEEEESS---SSS----SS-EEEEEEB--HHHHHHHSTTT--TTEEEES-TTSS-B--TT-TT-EEEETTEEEEEEEE-SSSSBT<br>TB-EEEEEGGGGHHHHHHHHHT-<br><br>IVGGYTCGANTKPYQVSLNSAYHFCGGSLPCSQWVVSAAHCYKSGNFDRLGEANINVVVGCEMFISASVSIVHQSYNSNTLNNDRMLIKLKSAAELNSRV<br>ASISLPTSCASAGTQMLISGWGNTKSSGTSYPDVLKCLKAPILSGSGCFSAYPGQITSNMFCAGYLEGGKDSCQGDSGGYVVCSGKLQGIVSWASGCAQK<br>NKPGVYTKVCNYVSWIKQTIASN<br><br>-BS-EE--TTS-TTEEEEEESSSEEEEEEEEEETTEEEE-GGG--TT-EEEES-SBTT---S--EEEEEEEEEE-TT--TTT-TT--EEEEESS----SSSS<br>---B--SS---TT-EEEEEESS---SSS----SB-EEEEEEB--HHHHHHHSTTT--TTEEEES-TT-S-B--TT-TT-EEEETTEEEEEEEE-SSSSBT<br>TB-EEEEEGGGGHHHHHHHHHH-	94.62%

(E)

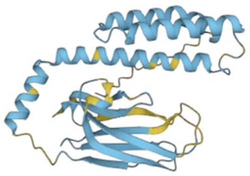

Alignment of condition, generated sequence and secondary structure	Concordance
-B-SS-EEEEETTTEEEEEEE-TT-BSSEEEEEEEEEESSTT--EEEEEEEE-S-SBGGGSEEEEEEEEEE------TT-S-EEEEEEE-TT--EEEEEEEETT<br>T--EEEEEE-TTTT---HHHHHHHHHHHHHTHHHHHHHHHHHHHHHHHHHHHHHHHHHHHHHH-TTTHHHH--TTHHHHHHHHHHHHHHHHHHT-TT--HHHHH<br>HHHHHHHHHHHHHHHHHHSS-SS---------EE--<br><br>DVHPLTLGTEDVEGAMIKLIHNRTVVQTSAYPNCSIVKDPQNTKRTSVYKGEIPLTKTVCLLGDFTLTGTPQGPNGVQQIEVTFETDARGILNVTADDKG<br>TGNKPKTTITNDQNILDPESIERMVNDAEKFAEEDKVGKEITDTANDLQDYKFKLKNQIGDKEKLGGKGSKEEKETMEKAVEEKIEMLESHPDADIEDFK<br>AKKKELEEIVFPRIKVLYGSEPLYFQGSNRLLLIG<br><br>-B-SS-EEEE-TTSBEEEEEE-TT-BSS-EE--EEE--STT--EEEEEEEE-SSSBGGGSEEEEEEEEEE------TT-EEEEEEEEE-TT--EEEEEEEETT<br>T----EEEE--TT----HHHHHHHHHHHHHTHHHHHHHHHHHHHHHHHHHHHHHHHHHHHHHH-TTTTTT-S-HHHHHHHHHHHHHHHHHHHHT-TT--HHHHH<br>HHHHHHHHHHHHHHHHHHH-S-------S-------	87.23%

(F)

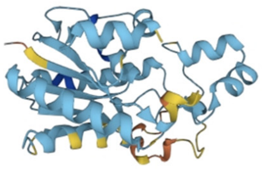

Alignment of condition, generated sequence and secondary structure	Concordance
--EEEEEEE---BHHHHTT---TT----B-HHHHHHHHHHHHHHHHHHTT---SEEEE-SSHHHHHHHHHHHHHHTT-TTS-EEE-GGGS----GGGTT--HH<br>HHHHHH-HHHHHHHHH-SS-------TTSTT--TT-GGGGGGTT--S---HHHHHHHHHHHIIIIIHHHHHTT--EEEEE-HHHHHHHHHHHTT--HHHH<br>HT----SS--EEEEE-TTS-BSSTT-EESSHHHHHHHHHH-<br><br>RTKDLTALRHGSSDWNANPGWTGWKDVALEPVGRGEAARSASLLVIHHLAQDLGYISGVRRLWTTAHLKLDTVDWGWIQVRRSWNLNSRHYGALQGLEKV<br>VTEARFGEERFWAMRRSYDTPPPQIEKGSDQSQTVDPRHTEIGGKQATDRLATVVTRFLQYYIDVIAPDLNTGRTVLIVACGNSLRALVKHLDEWSEDEV<br>VAAPKPTGINLIYDLDKDLRPVVQGLIYLDPEAAAAVISQ<br><br>--EEEEEEE---BHHHHTTBB-TT----B-HHHHHHHHHHHHHHHHHHTT---SEEEEESSHHHHHHHHHHHHHHTT-TTSEEEEEGGG-----GGGBT-BHH<br>HHHHHH-HHHHHHHHS-SS-------TT-TT-GGG-GGGGGGTT--S---HHHHHHHHHHHIIIIIHHHHHTT--EEEEE-HHHHHHHHHHHT--HHHH<br>HH----TT-EEEEEE-TTS-BSS-S--BSSHHHHHHHHHH-	90.83%

Figure S3. The generated results under last 3 conditions of 6 distinct conditions from **(D)** 1O2I A chain (223 residues), **(E)** 5E85 A chain (235 residues), and **(F)** 4EO9 A chain (240 residues). For each condition, the folding structures of the generated sequences with the highest conformity predicted by ESMFold (top panel in each sub-figures), and the alignments of the condition sequence, generated sequence, and corresponding secondary structure sequence (bottom panel in each sub-figures). In the top folding structures panel, the color of the residues represents the pLDDT Score range: dark blue for >90, light blue for 70-90, yellow for 50-70, and red for <50.

Table S1. The table provides information on 20 amino acids, including their abbreviations and the corresponding encoding method used in our model.

Name	Abbreviation	Encoding
Leucine	L	1
Glycine	G	2
Alanine	A	3
Valine	V	4
Lysine	K	5
Serine	S	6
Glutamic acid	E	7
Aspartic acid	D	8
Threonine	T	9
Isoleucine	I	10
Arginine	R	11
Asparagine	N	12
Proline	P	13
Glutamine	Q	14
Phenylalanine	F	15
Tyrosine	Y	16
Histidine	H	17
Cysteine	C	18
Methionine	M	19
Tryptophan	W	20

Table S2. The table displays the parameters or hyperparameters employed in PPDesigner, the revised Imagen model, the Denoiser, and the optimizer.

Component	Parameter or Hyperparameter	Value or Description
PPDesigner	Batch size	Varying from 96 to 256
	Max sequence length	250
	Dimension of input condition	1
	Dimension of output text embedding	256
	Probability of conditional dropout	0.1
Revised Imagen	Number of sample steps	64
	σ_{min}	0.002
	σ_{max}	80
	σ_{data}	0.5
	ρ	7
	P_{mean}	-1.2
	P_{std}	1.2
	S_{churn}	80
	$S_{t,min}$	0.05
	$S_{t,max}$	50
	S_{noise}	1.003
Denoiser	Number of LSTM's directions	2
	Number of LSTM's layers	5
	Dimension of LSTM input and output	1024
	Dimension of LSTM hidden and cell state	512
	Number of transformer encoder blocks	6
	Number of transformer decoder blocks	7
	Number of cross/self-attention heads	8
	Feed-forward in transformer encoder blocks	Input: 1024; hidden: 2048; output: 1024
	Feed-forward in transformer decoder blocks	Input: 1024; hidden: 1024; output: 1024
	Fully-connect blocks in Denoiser encoder	Input: 257; hidden: 1024; output: 1024
	Fully-connect blocks in transformer encoder	Input: 257; hidden: 512; output: 512
	θ in learnable ROPE	Learning during training process
	Dense blocks	Input: 1024; hidden: 512; output: 1; SELU
Adam optimizer	Learning rate	Varying from 1×10^{-4} to 1×10^{-7}
	ε	10^{-8}
	β	(0.9, 0.99)
